# Proportion and clinical features of never-smokers with non-small cell lung cancer

**DOI:** 10.1186/s40880-017-0187-6

**Published:** 2017-02-08

**Authors:** Jaeyoung Cho, Sun Mi Choi, Jinwoo Lee, Chang-Hoon Lee, Sang-Min Lee, Dong-Wan Kim, Jae-Joon Yim, Young Tae Kim, Chul-Gyu Yoo, Young Whan Kim, Sung Koo Han, Young Sik Park

**Affiliations:** 1 Department of Internal Medicine, Seoul National University Hospital, Seoul, 110-744 Republic of Korea; 2 Department of Thoracic and Cardiovascular Surgery, Seoul National University Hospital, Seoul, 110-744 Republic of Korea

**Keywords:** Non-small cell lung carcinoma, Never-smoker, Epidermal growth factor receptor, KRAS, Prognosis

## Abstract

**Background:**

The proportion of never-smokers with non-small cell lung cancer (NSCLC) is increasing, but that in Korea has not been well addressed in a large population. We aimed to evaluate the proportion and clinical features of never-smokers with NSCLC in a large single institution.

**Methods:**

We analyzed clinical data of 1860 consecutive patients who were newly diagnosed with NSCLC between June 2011 and December 2014.

**Results:**

Of the 1860 NSCLC patients, 707 (38.0%) were never-smokers. The proportions of women (83.7% vs. 5.6%) and adenocarcinoma (89.8% vs. 44.9%) were higher among never-smokers than among ever-smokers. Significantly more never-smokers were diagnosed at a younger median age (65 vs. 68 years, *P* < 0.001) and earlier stage (stage I–II, 44.5% vs. 38.9%, *P* = 0.015) compared with ever-smokers. Epidermal growth factor receptor mutations (57.8% vs. 24.4%, *P* < 0.001) and anaplastic lymphoma kinase rearrangements (7.8% vs. 2.8%, *P* < 0.001) were more common in never-smokers, whereas Kirsten rat sarcoma viral oncogene homolog mutations (5.8% vs. 9.6%, *P* = 0.021) were less frequently encountered in never-smokers than in ever-smokers. Never-smokers showed longer survival after adjusting for the favorable effects of younger age, female sex, adenocarcinoma histology, better performance status, early stage disease, being asymptomatic at diagnosis, received antitumor treatment, and the presence of driver mutations (hazard ratio, 0.624; 95% confidence interval, 0.460–0.848; *P* = 0.003).

**Conclusions:**

More than one-third of the Korean patients with NSCLC were never-smokers. NSCLC in never-smokers had different clinical characteristics and major driver mutations and resulted in longer overall survival compared with NSCLC in ever-smokers.

## Background

Lung cancer is the leading cause of cancer death, accounting for an estimated 1.6 million deaths in 2012 worldwide [1]. In Korea, lung cancer has been the most common cause of cancer death since 1999 and was expected to account for 22.6% of all cancer deaths in 2012, although the age-standardized mortality due to lung cancer has decreased slightly in both men and women since 2002 [2]. Lung cancer is considered a preventable cancer because tobacco smoking is the major cause of lung cancer [3]. According to comprehensive tobacco control programs, the incidence of lung cancer has been decreasing, whereas the proportion of never-smoking patients with non-small cell lung cancer (NSCLC) has been increasing [4, 5]. Global statistics estimate that overall 25% of lung cancers worldwide occur in never-smokers, and this proportion is approximately 10%–15% in Western countries [6]. The percentage is higher in Eastern Asia, as studies from Japan [5] and Singapore [7] have reported that never-smokers comprise approximately 32% of NSCLC patients.

It has been suggested that NSCLC in never-smokers should be regarded as a separate disease entity, as it bears striking differences from NSCLC in ever-smokers in terms of its epidemiologic and clinical characteristics; never-smokers with NSCLC are mostly women and have adenocarcinoma histology [5, 7, 8]. Recent molecular epidemiologic studies have supported that NSCLC in never-smokers has distinct underlying molecular mechanisms. Epidermal growth factor receptor (*EGFR*) mutations and anaplastic lymphoma kinase (*ALK*) rearrangements are more frequent in never-smokers with NSCLC, whereas Kirsten rat sarcoma viral oncogene homolog (*KRAS*) mutations are associated with ever-smokers [9–11]. These genetic alterations occur in a mutually exclusive fashion, providing evidence that NSCLC in never-smokers arises through different genetic pathways [8, 12].

Although there have been several studies reporting the clinical characteristics and prognosis of never-smoking NSCLC patients in Eastern Asia [5, 7], large-scale studies in the Korean population are lacking. This study was aimed to evaluate the proportion and clinical features of never-smokers with NSCLC in a large single institution in Korea.

## Patients and methods

### Patients

This study included all consecutive patients who were newly diagnosed with NSCLC at the Department of Internal Medicine, Seoul National University Hospital, Seoul, Republic of Korea between June 2011 and December 2014. Patients with recurred NSCLC were excluded. This study was approved by the institutional review board of the Seoul National University Hospital (H-1401-033-548). The requirement for informed consent was waived.

### Clinical and pathologic variables

All data were collected from medical records. Subjects were categorized based on the smoking status as never- and ever-smokers. An individual who had a lifetime exposure of <100 cigarettes was defined as a never-smoker. An individual who smoked no <100 cigarettes during one’s lifetime was defined as an ever-smoker. The clinical and pathologic data reviewed for analysis included age, sex, symptoms at diagnosis, histologic subtype, Eastern Cooperative Oncology Group (ECOG) performance status, disease stage, treatment received. Disease stage was defined as pathologic tumor, node, metastasis (TNM) stage for surgical cases and clinical TNM stage for non-surgical cases at the time of initial diagnosis, according to the seventh edition of the American Joint Committee on Cancer Staging Manual [13].

### Mutational analysis

DNA was extracted from formalin-fixed, paraffin-embedded tissue as per the standard protocol. Initially, the *EGFR* mutation status (exons 18–21) and the *KRAS* mutation status (exon 2) of the extracted DNA were determined with nested polymerase chain reaction (PCR) followed by bidirectional direct sequencing, as previously described [14]. However, after peptide nucleic acid (PNA) clamping technology was recognized as a more sensitive method compared to direct sequencing for the detection of gene mutations in diagnostic specimens with a low proportion of tumor cells [15], PNA-mediated real-time PCR clamping replaced direct sequencing since February 1, 2013. The PNAClamp *EGFR* Mutation Detection Kit (Panagene Inc., Daejeon, Korea) was used as previously described [15]. *KRAS* mutations in codons 12 and 13 were detected with PNA-mediated real-time PCR. To test for *ALK* rearrangements, immunohistochemistry (IHC) for ALK protein expression was used as a screening modality, and fluorescence in situ hybridization (FISH) was used for confirmation. Unstained slides of formalin-fixed, paraffin-embedded tumor tissues were analyzed with FISH using the Vysis *ALK* Dual Color, Break Apart Rearrangement probe (Abbott Molecular, Abbott Park, IL, USA) as previously described [16]. *ALK* rearrangements were defined via split *ALK* 5′ and 3′ probe signals or isolated 3′ signals in more than 15% of scored tumor cells [17].

### Follow-up

The schedules of follow-up were determined by clinicians. Overall survival (OS) was measured from the date of diagnosis to the date of death or the date of last follow-up. Death certificate data were obtained from the Korea National Statistical Office on 16 February 2015. The last follow-up was performed in February 2015.

### Statistical analysis

Continuous data are presented as median (range), whereas categorical data are presented as numbers (percentages). Clinicopathologic variables between never-smokers and ever-smokers were compared using the independent samples *t* test or the Mann–Whitney *U* test for continuous variables and the χ^2^ test or Fisher’s exact test for categorical variables. The OS rates were calculated according to the Kaplan–Meier method, and the differences among the groups were tested using the log-rank test. Multivariate analysis was performed with the Cox proportional hazards model adjusting for variables with a *P* < 0.2 in the univariate analysis. All statistical analyses were performed using Stata statistical software (Version 12.0, StataCorp LP, College Station, TX, USA).

## Results

### Clinical characteristics

A total of 1860 NSCLC patients diagnosed during the study period were selected. Of the 1860 patients, 707 (38.0%) were never-smokers, and 1153 (62.0%) were ever-smokers. The characteristics of 1860 NSCLC patients according to smoking status are listed in Table 1. The median age at diagnosis of never-smokers was significantly younger than that of ever-smokers (65 vs. 68 years, *P* < 0.001). A greater proportion of women were observed in the group of never-smokers than in the group of ever-smokers (*P* < 0.001), and the proportion of adenocarcinoma was higher in never-smokers than in ever-smokers (*P* < 0.001). Never-smokers had better ECOG performance status (*P* < 0.001), tended to present with earlier stage disease (stage I–II, 44.5% vs. 38.9%, *P* = 0.015), and were more likely to be asymptomatic than ever-smokers (*P* < 0.001).

**Table 1:** Characteristics of 1860 patients with NSCLC according to smoking status

Variable	Never-smokers [cases (%)]	Ever-smokers [cases (%)]	*P*
Total	707 (38.0)	1153 (62.0)	
Sex			<0.001
Men	115 (16.3)	1088 (94.4)	
Women	592 (83.7)	65 (5.6)	
Histologic subtype			<0.001
Adenocarcinoma	635 (89.8)	518 (44.9)	
Squamous cell carcinoma	25 (3.5)	483 (41.9)	
Others	47 (6.7)	152 (13.2)	
ECOG PS			<0.001
0	379 (53.6)	427 (37.0)	
1	269 (38.1)	558 (48.4)	
2	43 (6.1)	131 (11.4)	
3	15 (2.1)	31 (2.7)	
4	1 (0.1)	6 (0.5)	
Symptoms at diagnosis			<0.001
Asymptomatic	417 (59.0)	487 (42.2)	
Symptomatic	290 (41.0)	666 (57.8)	
Driver mutations			
*EGFR* mutations (*n* = 1284)	353 (57.8)	164 (24.4)	<0.001
*KRAS* mutations (*n* = 1089)	29 (5.8)	57 (9.6)	0.021
*ALK* rearrangements (*n* = 1288)	47 (7.8)	19 (2.8)	<0.001
Stage			<0.001
I	261 (36.9)	295 (25.6)	
II	54 (7.6)	153 (13.3)	
III	115 (16.3)	293 (25.4)	
IV	277 (39.2)	412 (35.7)	
Treatment			<0.001
Antitumor	654 (92.5)	983 (85.3)	
Supportive/unknown	53 (7.5)	170 (14.7)	

*NSCLC* non-small cell lung cancer, *ECOG* Eastern Cooperative Oncology Group, *PS* performance status, EGFR epidermal growth factor receptor, *KRAS* Kirsten rat sarcoma viral oncogene homolog, *ALK* anaplastic lymphoma kinase

### Frequencies of driver mutations according to smoking status

Of the 1860 patients, 1284 were tested for *EGFR* mutations, 1288 were tested for *ALK* rearrangements, and 1089 were tested for *KRAS* mutations. As expected, the frequencies of *EGFR* mutations (353/611 [57.8%] vs. 164/673 [24.4%]) and *ALK* rearrangements (47/605 [7.8%] vs. 19/683 [2.8%]) were significantly higher in never-smokers than in ever-smokers (both *P* < 0.001) (Table 1). Among the *EGFR* mutations, exon 19 deletions and exon 21 point mutations were significantly associated with never-smoking status (all *P* < 0.001), whereas mutations in exons 18 and 20 were not (*P* = 0.227, *P* = 0.167, respectively).

Of the 1089 NSCLC patients who were tested for *KRAS* mutations, 86 (7.9%) patients harbored a *KRAS* mutation. *KRAS* mutations were detected less frequently in never-smokers than in ever-smokers (29/497 [5.8%] vs. 57/592 [9.6%], *P* = 0.021) (Table 1). Of the 86 cases of *KRAS* mutations, 6 were detected with PNA clamping at codon 12; 80 were detected with direct sequencing (Table 2). Gly12Cys (28/80, 35.0%) was the most frequent *KRAS* mutation, followed by Gly12Asp (22/80, 27.5%) in Korean NSCLC patients. Of the 80 *KRAS* mutations, 27 (33.8%) were transition mutations. Gly12Asp was the most frequent *KRAS* mutation in never-smokers. The transition:transversion ratio of *KRAS* mutations was 12:15 for never-smokers and 15:38 for ever-smokers (*P* = 0.149).

**Table 2:** Type and frequency of *KRAS* mutations according to smoking status in 80 patients with NSCLC

*KRAS* mutation	Nucleotide substitution	Amino acid substitution	Never-smokers [cases (%)]	Ever-smokers [cases (%)]	Total [cases (%)]
Transversion	GGT>TGT	Gly12Cys	6 (22.2)	22 (41.5)	28 (35.0)
	GGT>GTT	Gly12Val	7 (25.9)	12 (22.6)	19 (23.8)
	GGT>GCT	Gly12Ala	2 (7.4)	3 (5.7)	5 (6.3)
	GGT>TTT	Gly12Phe	0	1 (1.9)	1 (1.3)
Transition	GGT>GAT	Gly12Asp	11 (40.7)	11 (20.8)	22 (27.5)
	GGT>AGT	Gly12Ser	0	2 (3.8)	2 (2.5)
	GGC>GAC	Gly13Asp	0	2 (3.8)	2 (2.5)
	CAG>CGG	Gln22Arg	1 (3.7)	0	1 (1.3)
Total			27	53	80

*KRAS* Kirsten rat sarcoma viral oncogene homolog, *NSCLC* non-small cell lung cancer

**Fig. 1:**
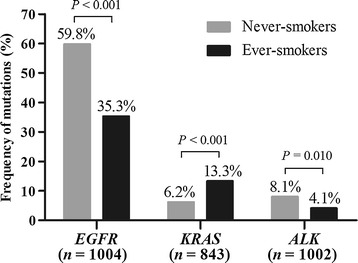
Frequencies of driver mutations in 1153 patients with lung adenocarcinoma according to smoking status. *EGFR* epidermal growth factor receptor, *KRAS* Kirsten rat sarcoma viral oncogene homolog, *ALK* anaplastic lymphoma kinase. *n* is the number of patients with lung adenocarcinoma who were tested for each mutation

In the subgroup of 1153 patients with lung adenocarcinoma, the frequencies of *EGFR* mutations (341/570 [59.8%] vs. 153/434 [35.3%], *P* < 0.001) and *ALK* rearrangements (46/566 [8.1%] vs. 18/436 [4.1%], *P* = 0.010) were significantly higher in never-smokers than in ever-smokers, whereas the frequency of *KRAS* mutations was significantly lower in never-smokers than in ever-smokers (29/466 [6.2%] vs. 50/377 [13.3%], *P* < 0.001) (Fig. 1).

### Survival analysis

**Fig. 2:**
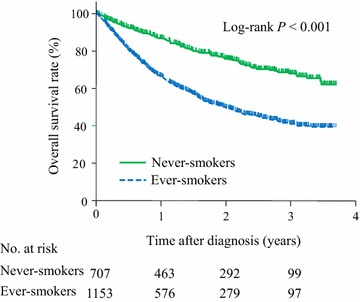
Kaplan-Meier overall survival curves of 1860 patients with non-small cell lung cancer (NSCLC) according to smoking status

The median follow-up time was 14.8 months (interquartile range 6.2–26.6 months). A total of 675 deaths (36.3%) occurred during follow-up. Never-smokers showed longer survival than ever-smokers (Fig. 2). The median OS were not yet reached for never-smokers and was 23.9 months for ever-smokers (95% CI 19.9–27.0 months). The 2-year OS rates were 75.8% for never-smokers and 49.8% for ever-smokers (*P* < 0.001).

In addition to smoking status, the prognostic factors determined by univariate analyses were age, sex, histologic subtype, ECOG performance status, disease stage, the presence of symptoms at diagnosis, *EGFR* mutations, *KRAS* mutations, *ALK* rearrangements, and treatment received. A multivariate analysis was performed with the Cox proportional hazards model including those factors, in which never-smoking status was associated with prolonged OS (hazard ratio [HR] 0.624; 95% CI 0.460–0.848; *P* = 0.003) (Table 3). Younger age, adenocarcinoma histology, better performance status, earlier stage, being asymptomatic at diagnosis, and antitumor treatment were also independent favorable prognostic factors. *EGFR* mutations (HR 0.493; 95% CI 0.381–0.637; *P* < 0.001) and *ALK* rearrangements (HR 0.397; 95% CI 0.239–0.660; *P* < 0.001) were associated with prolonged OS, whereas *KRAS* mutations were associated with an increased risk of death (HR 1.560; 95% CI 1.085–2.243; *P* = 0.017).

**Table 3:** Multivariate analysis of overall survival using the Cox regression in 1860 patients with NSCLC

Variable	Whole cohort (*n* = 1860)			Never-smokers (*n* = 707)			Ever-smokers (*n* = 1153)		
	HR	95% CI	*P*	HR	95% CI	*P*	HR	95% CI	*P*
Smoking status				NA			NA		
Ever-smoker	1.000								
Never-smoker	0.624	0.460–0.848	0.003						
Age^a^	1.023	1.014–1.032	<0.001	1.005	0.988–1.021	0.582	1.029	1.018–1.040	<0.001
Sex									
Male	1.000			1.000			1.000		
Female	1.054	0.780–1.426	0.731	0.977	0.619–1.544	0.922	1.179	0.794–1.750	0.414
Histologic subtype									
Adenocarcinoma	1.000			1.000			1.000		
Squamous cell carcinoma	1.387	1.115–1.727	0.003	2.262	1.131–4.523	0.021	1.295	1.019–1.645	0.035
Others	1.506	1.184–1.915	0.001	1.066	0.618–1.839	0.818	1.586	1.201–2.095	0.001
ECOG PS									
0–1	1.000			1.000			1.000		
2–4	2.098	1.725–2.552	<0.001	2.743	1.732–4.343	<0.001	1.976	1.589–2.457	<0.001
Symptoms at diagnosis									
Asymptomatic	1.000			1.000			1.000		
Symptomatic	0.722	0.610–0.854	<0.001	0.715	0.503–1.015	0.061	0.737	0.608–0.894	0.002
*EGFR* mutations^b^									
Absent	1.000			1.000			1.000		
Present	0.493	0.381–0.637	<0.001	0.516	0.352–0.757	0.001	0.459	0.314–0.671	<0.001
*KRAS* mutations^b^									
Absent	1.000			1.000			1.000		
Present	1.560	1.085–2.243	0.017	2.540	1.253–5.150	0.010	1.416	0.921–2.176	0.113
*ALK* rearrangements^b^									
Absent	1.000			1.000			1.000		
Present	0.397	0.239–0.660	<0.001	0.225	0.105–0.484	<0.001	0.723	0.362–1.444	0.358
Stage									
I	1.000			1.000			1.000		
II	2.087	1.387–3.143	<0.001	3.995	1.365–11.694	0.011	1.810	1.163–2.818	0.009
III	4.323	3.095–6.039	<0.001	4.470	1.872–10.671	0.001	4.036	2.797–5.824	<0.001
IV	10.314	7.507–14.172	<0.001	23.067	10.840–49.085	<0.001	8.437	5.908–12.049	<0.001
Treatment									
Supportive/unknown	1.000			1.000			1.000		
Antitumor	0.634	0.516–0.780	<0.001	0.377	0.214–0.662	0.001	0.678	0.541–0.849	0.001

*NSCLC* non-small cell lung cancer, *HR* hazard ratio, *CI* confidence interval, *NA* not applicable, *ECOG* Eastern Cooperative Oncology Group, *PS* performance status, *EGFR* epidermal growth factor receptor, *KRAS* Kirsten rat sarcoma viral oncogene homolog, *ALK* anaplastic lymphoma kinase

^a^Age was used as a continuous variable

^b^The individuals who did not undergo testing for each mutation were included in the multivariate analysis, however, the data are not shown

Separate multivariate survival analyses were further conducted for 707 never-smokers and 1153 ever-smokers (Table 3). The histologic subtype, ECOG performance status, disease stage, treatment received, and status of *EGFR* mutations were common prognostic factors in both never- and ever-smokers. However, younger age and being asymptomatic at diagnosis were no longer associated with prolonged OS in never-smokers. In ever-smokers, *KRAS* mutations and *ALK* rearrangements were not independent prognostic factors for OS.

## Discussion

In the current study, we summarized the clinical characteristics and major driver mutations of NSCLC patients and compared survival outcomes between never- and ever-smokers in 1860 Korean NSCLC patients. We provided the profile of major genes (*EGFR*, *KRAS*, and *ALK*), and we adjusted for the presence of each driver mutation in the survival analysis. Never-smokers accounted for 38.0% of the NSCLC patients, and smoking status was an independent prognostic factor regardless of whether NSCLC patients had oncogenic driver mutations.

In our study, never-smokers were diagnosed at an earlier age than ever-smokers, which was consistent with a previous report from Singapore [7]. However, several studies from Western countries reported the opposite observation: never-smokers were diagnosed at a similar or older age than ever-smokers [4, 18]. This might be explained by later ages of initiation of smoking in Asian smokers than in Western smokers; because the age at smoking onset in Asian countries is much older than that in Western countries, the age at cancer diagnosis for Asian smokers may be older. In addition, the great contribution of risk factors other than smoking might affect the development of lung cancer in Asian countries [19]. Another interesting observation was that never-smokers tended to present disease at an early stage. This finding was consistent with the result of a study performed in Japan [5], whereas studies from Singapore [7] and the United States [20] reported that never-smokers were more likely to present with advanced disease and another study from the United States found no differences [18].

Although *EGFR* mutations have been well known as the most common genetic alteration in never-smoking NSCLC patients, *EGFR* mutations were detected in 24.4% of ever-smoking NSCLC patients in our study. The frequencies of *EGFR* mutations in ever-smokers vary widely, from 8.4% to 11.9% in Europe and North America to 26.0%–35.9% in Japan and China [21]. Several potential risk factors other than *EGFR* mutations in never-smokers have been proposed, including environmental tobacco smoke, residential radon, asbestos exposure, cooking oil fumes, genetic susceptibility, hormone factors, and oncogenic viruses, which may contribute with varied degrees in line with sex and geographical areas [8].

In the present study, Gly12Cys, resulting from a codon 12 GGT>TGT substitution, was the most frequent subtype of *KRAS* mutations, which was consistent with the results of studies in Western countries [22, 23]. However, a recent study analyzing 82 *KRAS* mutations in 1420 Korean NSCLC patients reported that Gly12Asp, resulting from a codon 12 GGT>GAT substitution, was the most frequent *KRAS* substitution [24]. In contrast to the previous studies showing that never-smokers were more likely to have a transition mutation rather than a transversion mutation [22, 24], the association between never-smoking status and transition mutations was not statistically significant in our cohort (*P* = 0.149). Because the frequency of *KRAS* mutations in the Asian population is relatively low, our study lacked the power to reach any firm conclusions about distinct characteristics of *KRAS* mutation in the Korean population even though the study included over one thousand NSCLC patients who were tested for *KRAS* mutations.

Our study has several limitations, such as its retrospective design, and the fact that it was conducted at a single institution. However, our institution is a 1750-bed tertiary teaching hospital and a large referral center in Korea. Moreover, we recruited all consecutive patients with newly diagnosed NSCLC during the study period to minimize the selection bias. The major limitation of this study was that the information regarding administration of specific targeted agents was not available. The use of targeted therapy may be a confounding factor of inherent mutation status in predicting survival. Another limitation was the relatively short follow-up period. In addition, information about exposure to risk factors other than smoking was unavailable. Further studies are needed to elucidate the potential risk factors for NSCLC in never-smokers.

## Conclusions

In summary, more than one-third of Korean patients with NSCLC were never-smokers. We showed distinct features of NSCLC in Korean never-smokers compared with ever-smokers, including a better prognosis in never-smokers. After adjusting for the favorable effects of younger age, better performance status, earlier stage of disease, being asymptomatic at diagnosis, and the presence of driver mutations, the association between never-smoking status and prolonged survival became apparent.
